# Twenty-five Cases of Tetanus

**Published:** 1918-01

**Authors:** 


***Twenty-five Cases of Tetanus.*** (A Report to the Medical Research Committee). By H. R. DEAN, Major, R. A. M. C. (T.). Abs. from *Lancet*, May 5, 1917.

D. reports with considerable detail 25 cases of tetanus seen in the 2nd Western General Hospital, Manchester, England. Most of the cases had probably received a preventive inoculation in France. All the wounds had been septic, but 9 of them had healed before tetanus set in. 13 of the wounds were severe. 5 cases of tetanus; however, occurred in very slight wounds.

L. notes that compound fractures were especially liable to tetanus. He finds that the preventive inoculation tends greatly to prolong the incubation period and also to prolong the period of onset, although he also notes cases in which the onset was exceedingly abrupt with spasms of the muscles, jaw, and neck. After a period of localization, varying considerably in length, general stiffening of the jaw, neck, and abdomen occurred.

As to the method of injection, D. concludes that the intramuscular route affords sufficient curative action if the signs are well localized. If the signs are generalized he thinks the intravenous method, in spite of all that has been said of its dangers, is much the best. He administers 30,000 units under deep chloroform anesthesia. This dose usually arrests all progress of the disease, and effects a cure within from 2 to 7 days. Out of 14 cases so treated, 13 recovered. In 7 cases in which the intravenous method was not accompanied by any other, the recovery was rapid and complete. There was free antitoxin in the serum of patients up to 39 days after the intravenous injections.

